# Implication of Sulfatide in Type 1 Diabetes and Multiple Sclerosis. Comment on Root-Bernstein, R. Type 1 Diabetes-Associated Autoantibodies, Sera and T-Cell Receptor Sequences Recognize Myelin Basic Protein: A Novel Peripheral Diabetic Neuropathy–Multiple Sclerosis Connection. *Cells* 2026, *15*, 1384

**DOI:** 10.3390/cells15181635

**Published:** 2026-09-10

**Authors:** Rikke Thea, Karsten Buschard

**Affiliations:** Faculty of Health and Medical Sciences, University of Copenhagen, DK-1870 Copenhagen, Denmark; rtl@sund.ku.dk

An interesting article by Root-Bernstein [1] has recently been published in *Cells* describing a potential immunological link between multiple sclerosis and type 1 diabetes through cross-reactivity involving myelin basic protein. It fits well with earlier findings indicating that multiple sclerosis is three times more frequent in patients with type 1 diabetes [2]. We would like to draw attention to another molecule that is highly relevant to both diseases, namely the sphingolipid sulfatide. Sphingolipids are special molecules which consist of fat chains, the amino acid serine, a galactose group and, for sulfatide, a sulfate group [3]. This molecule is enriched in both the myelin sheath of the central nervous system and the pancreatic islets of Langerhans. Interestingly, sulfatide can inhibit T lymphocyte activity [4], a process of high relevance to autoimmune diseases such as multiple sclerosis and type 1 diabetes. Furthermore, antibodies directed against sulfatide exist both in type 1 diabetes [5] and multiple sclerosis [6], reducing the protective immunoregulatory effect of this sphingolipid. Both pancreatic islets and neural tissue are susceptible to viral infection, and sulfatide has been implicated in virus binding, with viruses using sulfatide as a receptor and potentially increasing the level of anti-sulfatide antibodies [7]. Thus, the sphingolipid sulfatide is a common molecule between the neural tissue and islets of Langerhans, and functional depletion of sulfatide by antibodies seems to be important for both type 1 diabetes and multiple sclerosis [8]. The role of sulfatide may be as important as that of myelin basic protein. Regarding prophylaxis or early treatment, it should be mentioned that both serine supplementation and fenofibrate, which enhances sulfatide levels, should be considered [9].

## Data Availability

No new data were created or analyzed in this study. Data sharing is not applicable to this article.
